# Efficacy and safety of combined oral sucrose and nonnutritive sucking in pain management for infants: A systematic review and meta-analysis

**DOI:** 10.1371/journal.pone.0268033

**Published:** 2022-05-06

**Authors:** Qiaohong Li, Xuerong Tan, Xueqing Li, Wenxiu Tang, Lin Mei, Gang Cheng, Yongrong Zou

**Affiliations:** 1 Department of Neonatology, Ya’an People’s Hospital, Ya’an, Sichuan, China; 2 Department of Pediatrics, Ya’an People’s Hospital, Ya’an, Sichuan, China; All India Institute of Medical Science - Bhopal, INDIA

## Abstract

**Background:**

Pain management is currently important in neonatal intensive care unit (NICU). The superiority in pain relief of the combined oral sucrose (OS) and nonnutritive sucking (NNS) to other single intervention has not been well established. The administration of sucrose has been considered to potentially induce adverse events, which has been controversial. This study aims to investigate the combined effects and safety in comparison with other single intervention methods, including NNS, OS alone, breast milk and oral glucose.

**Methods:**

We searched databases including Medline (via Pubmed), Embase (via Ovid), web of science, and Cochrane Library for randomized controlled trials from Jan 1, 2000 to Mar 31, 2021. The data were analyzed in the meta-analysis using Review manager Version 5.3. Pain score was the primary outcome in this meta-analysis. The adverse events were assessed qualitatively.

**Results:**

A total of 16 studies were eligible in the meta-analysis. The results demonstrated a significant reduction in pain score in the NNS+OS group compared with NNS alone (SMD = -1.69, 95%CI, -1.69,-0.65) or sucrose alone (SMD = -1.39, 95% CI, -2.21,-0.57) during the painful procedures. When compared NNS+OS with breast milk, no significant difference was detected (SMD = -0.19, 95% CI: -0.5, 0.11).

**Conclusion:**

The combined effects of NNS and OS might be superior to other single intervention method. However, the effects might be mild for moderate-to-severe pain.

## 1 Introduction

Newborns, especially preterm infants, are frequently subjected to painful procedures [1], repeated painful and stressful stimuli may develop clinical, physiologic and psychologic sequelae in the short or long term [2–4]. In some developing countries, most infants were not provided any analgesic intervention during painful procedures [5, 6]. Even in a developed country like Canada, analgesic interventions were not offered in almost half of painful procedures, according to an epidemiology study [7]. In addition, it remains unclear whether the administration of anesthetics is safe [8]; thus, selective use of pharmacological treatment is recommended, such as opioids, non-opioids, and other anesthetics [9]. On the other hand, nonpharmacological methods have been developed in recent years to help reduce multiple bedside interruptions and have been considered safe and effective in pain management [10, 11]. An updated research revealed the use rate of nonpharmacologic interventions tended to be higher than pharmacologic interventions [12].

Combined therapy was assumed to be more efficient in pain relief than single interventions and has been investigated frequently in recent studies [13–16]. For example, combined effects of music and touch [17], sucking, breast milk and tucking [18], music and sucrose [19], oral sucrose (OS) and nonnutritive sucking (NNS) [20, 21], etc. were evaluated in clinical trials. Among which the combined effects of NNS and sucrose were tested by numerous prospective randomized trials. It has been common knowledge that OS and NNS could separately alleviated pain in neonates. Previous reviews or meta-analyses have preliminarily evaluated the combined effects of NNS and sucrose [10, 13]. However, they may have neglected the variations in effectiveness and safety under different painful procedures. The superiority of the combination to various single interventions was also barely covered [8]. Therefore, this systematic review and meta-analysis aim to fill the gap by evaluating the efficacy of combining OS and NNS in different scenarios of comparison and exploring the safety of this intervention method preliminarily.

## 2 Methods

### 2.1 Literature search and screen

We conducted a systematic search from the following databases for English language articles: Medline (via PubMed), Embase (via Ovid), Cochrane Library, Web of Science. The search terms were based on three domains “newborns”, “sucrose and non-nutritive sucking” and “procedural pain”. The outcome-related terms were not restricted. Randomized controlled trials were selected using database-specific limiters. The time span of publication years was limited from Jan 1, 2000 to Mar 31, 2021. The search strategies for each database were provided (S1 Table). In addition to the electronic searches, Google Scholar and references in literature were also manually searched for potential suitable studies. The unpublished studies were not considered. The language restriction was English.

The literature research and screening procedures afterwards were performed independently by two researchers. The final eligible studies were cross-checked. If there were any disagreements, a third author made judgments.

### 2.2 Inclusion and exclusion criteria

#### 2.2.1 Inclusion criteria

(1) The study was a randomized controlled trial. (2) The paticipants were preterm or full term neonates without severe illness. (3) The randomized groups in the trial should at least contain an intervention group applied with OS combined with NNS. (4) Conference abstracts eligible for the inclusion criteria above were also included.

#### 2.2.2 Exclusion criteria

(1) Results of pain score were presented as categorical outcome. (2) The sample size, mean and standard deviation were partly provided and could not be estimated from other statistics such as mean difference and p-value or median, range and interquartile range. (3) Non-English literature was excluded. (4) The outcomes of no interest, e.g., the occurrence of startle, jerk or tremor were excluded.

### 2.3 Literature quality evaluation

We conducted literature quality evaluation according to the Cochrane Risk of Bias Tool (RoB), version 5.2 [22]. The risk of bias was assessed on a 3 level scale:”low”, “high” and “unclear” risk based on the following seven domains: random sequence generation, allocation concealment, blinding of participants and personnel, blinding of outcome assessment, incomplete outcome data, selective reporting, intention-to-treat analysis, and a completeness of follow-up.

### 2.4 Data extraction

Data extraction was performed by two reviewers independently. The summary of studies was established from the following information: first author, year of publication, country, study design, gestational age, pain procedure, sample size and specific intervention descriptions in each group and outcome.

We implemented the meta-analysis on pain score measured in different painful procedures, including Premature Infant Pain Profile(PIPP) [23], the premature infant pain profile-revised (PIPP-R) [24], Neonatal Infant Pain Scale (NIPS) [25], the Neonatal Facial Coding System(NFCS) [26] Neonatal Pain Agitation and Sedation Scale (N-PASS) [27]. The results of pain score were required to be presented as continuous variables. The Means and standard deviations (SDs) were extracted directly from the original data if available. Otherwise, they were estimated using Hozo et al.’s Method or Bland’s Method under the scenario where median, IQR or ranges were available [28–30]. Calculations using p-value, confidence interval and mean difference were performed following the instructions in Cochrane handbook 5.1 [22]. Statistics were estimated from figures if applicable when no digits were provided. Missing data were not imputed. For cross-over designs trials, paired analysis was performed to standardize the means and SDs to account for the within-subject correlation [31]. If no information about within-subject correlation was provided, we decided to assume the correlation to be 0 which was a conservative way since there were no similar data to be referred in the included studies.

For evaluating the effects at different timepoints, we extracted the outcome data in 2 phases, (1) during the procedure phase (during or within the first minute immediately after the painful procedure), and (2) the recovery phase (1 to 5 min after the procedure). Pairwise comparisons were performed between the intervention group of interest, i.e., sucrose and NNS group and any other relevant groups including NNS (NNS+water)/sucrose alone, breastfeeding, breast milk, glucose or routine care group. In crossover trials, the overall effects were extracted instead of looking at a single sequence.

### 2.5 Statistical analysis

Results of pain score were presented as standardized mean difference (SMD) if holding different scales and 95% CI considering variety among pain measurements. If studies shared the same measurement then mean difference (MD) could be presented. A p-value <0.05 was considered as significant. Statistical heterogeneity was assessed using I^2^ tests. Subgroup analysis or random-effects model applied when the statistical heterogeneity was high (I^2^ >50%) [22]. Otherwise, a fixed-effects model was conducted. Sensitivity analyses were conducted to assess the stability and validity of results. Factors were considered when removing certain studies, including article types, outcome measurements (PIPP or non-PIPP), target population (preterm/full term), etc. Publication bias was evaluated by Egger’s Test and funnel plots. All the statistical analysis was performed using Review Manager 5.3 [32]. Egger’s Test was performed using SAS version 9.4. Besides, the safety of the intervention was qualitatively evaluated based on the occurrence and types of adverse events.

## 3 Results

### 3.1 Outline of eligible studies

**Fig 1:**
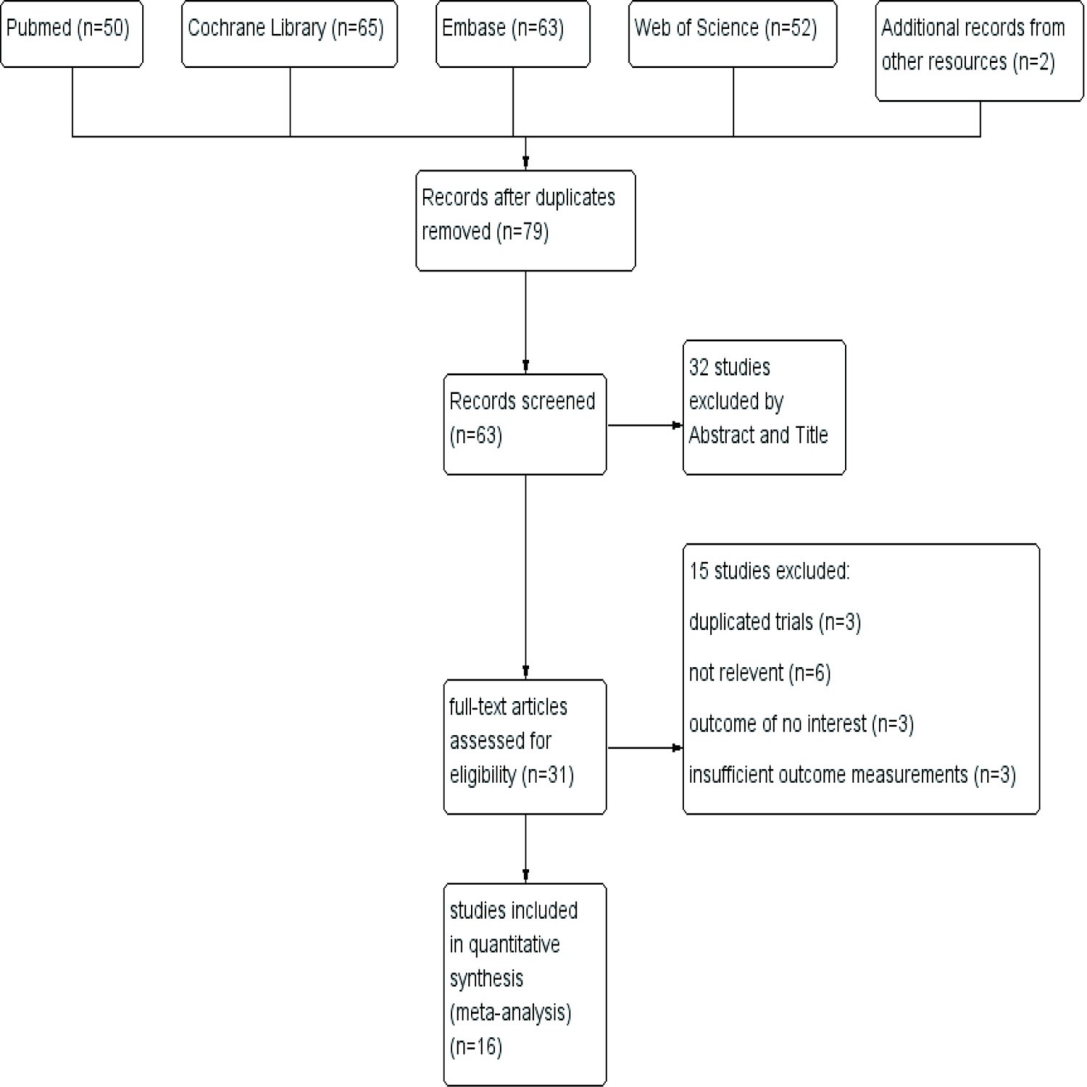
Flow diagram of study inclusion and exclusion.

A total of 232 records were identified using our searching strategy from databases and two from the references in a previous review [10]. After removing duplicates, 79 records were obtained and 63 of them were screened in the first stage by looking through abstracts and titles. The first round of screening led to 31 records for the following full-text review. Finally, 16 studies [33–40, 42–49] were included in our systematic review and meta-analysis. The screening process was described as the PRISMA flow diagram (Fig 1).

**Fig 2:**
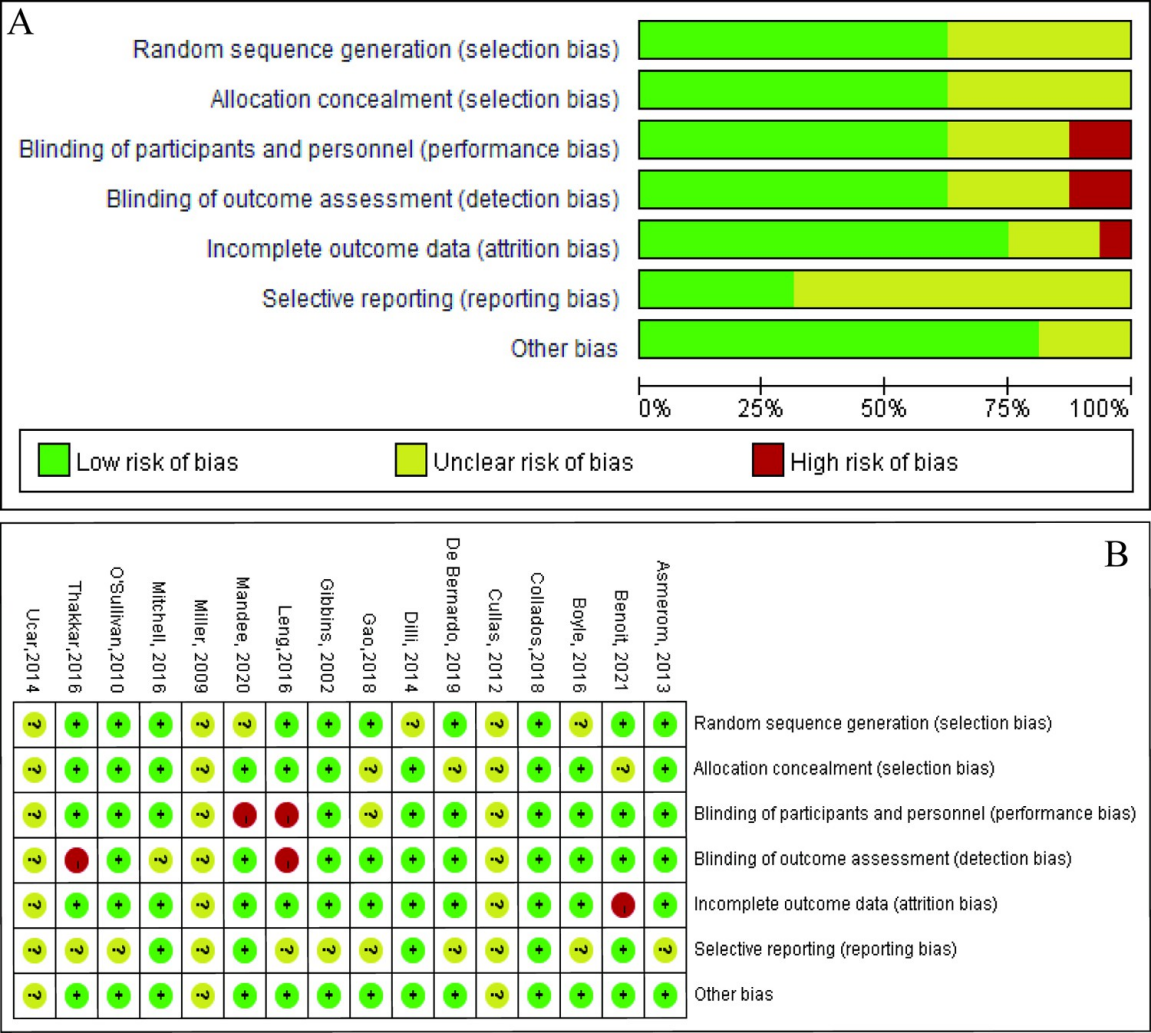
Risk of bias assessment for each included trial. A, Risk of bias graph; B: Risk of bias summary.

There were three conference abstracts among included studies [33–35]. Of all the RCTs included, two were based on a cross-over design [34, 36]. Eligible Infants enrolled were categorized by preterm (n = 8), full-term (n = 5) and both (n = 3). Infants underwent different painful procedures including heel stick (n = 8), retinopathy of prematurity (ROP) screening (n = 5), venipuncture (n = 2) and wound dressing (n = 1). PIPP score was mostly widely used for pain assessment (n = 10) while PIPP-R(n = 1), NIPS (n = 3), NFCS (n = 1) and N-pass score(n = 1) were also applied in other studies. Characteristics of eligible studies were outlined in Table 1. Fig 2A, 2B demonstrated the risk of bias assessment for each study.

**Table 1:** The characteristics of included studies.

First author, year, country	Study Type/design	Objects	Procedure	Sample size	Gestational age and weight (inclusion criteria)	Intervention/Control groups	Outcomes
Asmerom,2013, United States [46]	Prospective double-blind randomized controlled study	preterm	heel lance	sucrose+NNS group: n = 44	premature infants ≤ 36.5	sucrose+NNS group:	PIPP scored from the time of heel lance to 30 seconds post the lance.
						received a single dose of 24% sucrose via syringe to the anterior tongue along with a pacifier (NNS) two minutes before the heel lance.	
				water+NNS group: n = 45	weeks gestation who weighed ≥800 grams		
						Water+NNS group: the placebo group received an equal volume of sterile water to the anterior portion of the tongue along with a pacifier.	
Benoit,2021, Canada [47]	Single-blind randomized controlled trial	healthy full-term	heel lance	sucrose+NNS: n = 19	healthy, full-term (born ≥ 37 0/7 weeks’gestation)	Intervention group: 24% oral sucrose combined with offered NNS and containment in a blanket while in an infant cot. Control group: direct breastfeeding	1.pain-related brain activity
							2.PIPP-R at 30-, 60-, 90-, and 120-s following heel lance
				breastfeeding: n = 18			
							3.adverse events
Boyle, 2016, Canada [42]	Prospective, Randomised, placebo controlled study	preterm	ROP screening	group 1: n = 10	< 32 weeks’ PMA	Group 1: 1 ml sterile water given by mouth using a syringe	PIPP scores during eye examination
				group 2: n = 10			
						Group 2: 1 ml sucrose 33% given by mouth using a syringe	
				group 3: n = 9		Group 3: 1 ml sterile water given by mouth using a syringe and pacifier put into the mouth	
				group 4: n = 11		Group 4: 1 ml sucrose 33% given by mouth using a syringe and pacifier put into the mouth	
Collados,2018, Spain [36]	Multicentre randomised, non-inferiority, cross-over trial	preterm	venipuncture	EBM-sucrose sequence: n = 33	gestational age of less than 37 weeks at birth and weigh less than 2,500 grams.	In group one, the neonate received EBM during the first venipuncture that was included in the study and 24% sucrose during the second venipuncture. The process was reversed for group two.	PIPP, the duration of crying, oxygen saturation and heart rate
				sucrose-EBM sequence: n = 33			
						This accompanied throughout by non-nutritive sucking and swaddling.	
Cullas,2012, Turkey [33]	Prospective randomised study	preterm	ROP screening	sucrose+NNS: n = 21	patients under 32 weeks of gestational age	Group 1: oral sucrose solution given two minutes before examination. Pacifier was used.	PIPP, time of crying
				water+NNS: n = 19			
						Group 2: sterile water given two minutes before examination. Pacifier was used.	
De Bernardo,2019, Italy [48]	Randomized double-blinded case–control pilot study	full-term	venipuncture	sucrose+NNS: n = 33	neonates 37–42 weeks gestational age at birth and >1 week old at the time of the intervention with body weight 2,500–4,500 g and able to feed orally.	Intervention group: received both 1 mL 24% sucrose orally via syringe 1 minute before venipuncture and 1 mL during the procedure. A pacifier was offered to all neonates immediately following sucrose administration each infant.	NIPS
							Outcome measurements (HR, SpO2) were obtained before (T0), during (T1), and 1 minute after (T2) venipuncture
				glucose+NNS: n = 33			
						Control: received 1 mL 10% glucose orally via syringe with a pacifier 1 minute before venipuncture and during the procedure	
Dilli, 2014, Turkey [49]	Prospective randomised and placebo-controlled study	preterm	ROP screening	Group 1: n = 32	-	Group 1: 0.5 mL/kg of sucrose 24% was given by mouth using a syringe, and pacifier was placed in the mouth.	PIPP score during examination crying time
				Group 2: n = 32			
						Group 2: 0.5 mL/kg of sterile water was given by mouth using a syringe, and pacifier was placed in the mouth.	
Gao,2018, China [44]	Randomized controlled trial	Preterm infants	heel stick	1.NNS group: n = 22	before 37 weeks of gestation	1.NNS group: pacifier given in 2 minutes before, and throughout the recovery phase of the heel stick.	1.PIPP scale in the blood collection phase (0-60s) and recovery phase (after 1 min)
						2. sucrose group: Sucrose 20% (0.2 mL/kg) was administrated to the preterm infant’s mouth by 1 ml syringe in 2 minutes before the heel stick procedure	2.heart rate and oxygen saturation
							3.the percentage of crying time respectively in the blood collection phase and recovery phase
						3.sucrose+NNS: Sucrose 20% (0.2 mL/kg) was administrated to the preterm infant’s mouth by 1 ml syringe in 2 minutes before the heel stick procedure and a pacifier was given until the recovery phase of the heel stick.	

						4. routine care group: received only routine comfort through gentle touch when he cried after the heel stick procedure.	
				2.sucrose group: n = 21			
				3.NNS+sucrose group: n = 22			
				4.routine care group: n = 21			
Gibbins,2002, Canada [45]	Randomized controlled trial	Preterm/term	heel lance	1.sucrose+NNS: n = 64	born between 27 and 43 weeks gestation	1. sucrose+NNS group: received 0.5 ml of 24% sucrose via a syringe followed immediately by the insertion of a pacifier into the mouth. The pacifier was held in place as required 2 minutes before, during, and 5 minutes following the heel lance.	the PIPP are numerically scored on 30/60 seconds following an acute painful stimulus.
				2.sucrose alone: n = 62			
				3.water+NNS: n = 64			
						2. sucrose group: received 0.5 ml of 24% sucrose via a syringe.	
						3.water+NNS group: received 0.5 ml of sterile water via a syringe. No pacifier was offered.	
Leng,2016, China [43]	Prospective, multi-centred, randomized controlled clinical trial	full term	shallow or deep heel stick procedures	NS group: n = 167	gestational age between 37 and 42 weeks at delivery;	Group S: 2 ml of 24% sucrose was administrated by syringe 2 min before the heelstick procedure.	NFCS score
						Group NS: 2 ml of 24% sucrose was administrated by syringe 2 min before the heel stick procedure, and then a standard silicone newborn pacifier was placed into the infant’s mouth until the end of the process.	
				S group: n = 176			
					Birthweight between 2500 g and 4000 g;		
Miller, 2009, United States [34]	Repeated-measures crossover design.	preterm/full term	heel stick	NNS+sucrose-no treatment sequence: n = 7	between the ages of 32	1.In the treatment condition, infants were offered NNS with sucrose.	NIPS score, heart rate and oxygen saturation
				no treatment-NNS+sucrose sequence: n = 7	weeks to younger than or equal to 42 weeks	2.In the control condition, infants were not offered any treatment	
Mandee,2020, Thailand [37]	Prospective randomized control trial	preterm/full term	Wound dressing	sucrose+NNS group: n = 16	-	Sucrose+NNS group: participants were first administered a dose of 24% sucrose and then were given the pacifier.	The NIPS scores were assessed at 30, 120, and 240 seconds
						NNS group: participants were administered a pacifier while their wound dressing was performed.	from the commencement of the wound dressing, crying time
				NNS group: n = 16			
Mitchell,2016, United States [39]	Double-blind 2×2 factorial randomized controlled trial	full term	heel stick	sucrose+NNS group: n = 37	healthy term infants between 37 and 42 weeks	1.sucrose+NNS: received 1±0.1 ml of the 24% sucrose solution orally with pacifier at 2 ±0.5 minutes prior to the procedure 2.water+NNS: received 1 ±0.1 ml of sterile water with pacifier	PIPP, heart rate variability (HRV), and salivary cortisol
				water+NNS group: n = 39			
O’Sullivan,2010, Ireland [38]	Randomised placebo controlled study	preterm	ROP screening	sucrose+NNS group: n = 20	< 1501 g	1. sucrose+NNS: infants were swaddled and received 0.2 ml of sucrose 24% given by mouth using a syringe and a soother	N-PASS score
				water+NNS group: n = 20	< 32 Weeks gestation Infants		
						2. water+NNS: infantsswaddled, and received 0.2 ml of sterile water given by mouth using a syringe and a soother.	
Thakkar,2016, India [40]	Randomized controlled trial	full term	heel-stick	group I (sucrose): n = 45	(>37 weeks PMA), with birthweight > 2200 g	1.group I received 30% sucrose solution by sterile syringe;	PIPP score, heart rate, oxygen saturation, duration of crying
				group II (NNS): n = 45			
						2.group II received NNS in which sterile gauze was held gently in neonate’s mouth and the palate tickled to stimulate sucking;	
				group III (sucrose+NNS): n = 45			
						3.group III received both the interventions (sucrose and NNS);	
						4.group IV received no intervention.	
				group IV: n = 45			
Ucar S,2014, Turkey [35]	Randomised, controlled study	preterm	prematurity (ROP) screening	sucrose group: n = 27	-	group 1 received 24% sucrose oral,	PIPP
						group 2 received 24% sucrose with pacifier	
				sucrose+NNS: n = 27			
				water+NNS: n = 27			
						group 3 received sterile water with pacifier.	

ROP: retinopathy of prematurity; NNS: nonnutritive sucking; PIPP: Premature Infant Pain Profile; NFCS: Neonatal Facial Coding System; NIPS: Neonatal Infant Pain Scale

### 3.2 Pain score

#### 3.2.1 NNS+OS group versus NNS group

**Fig 3:**
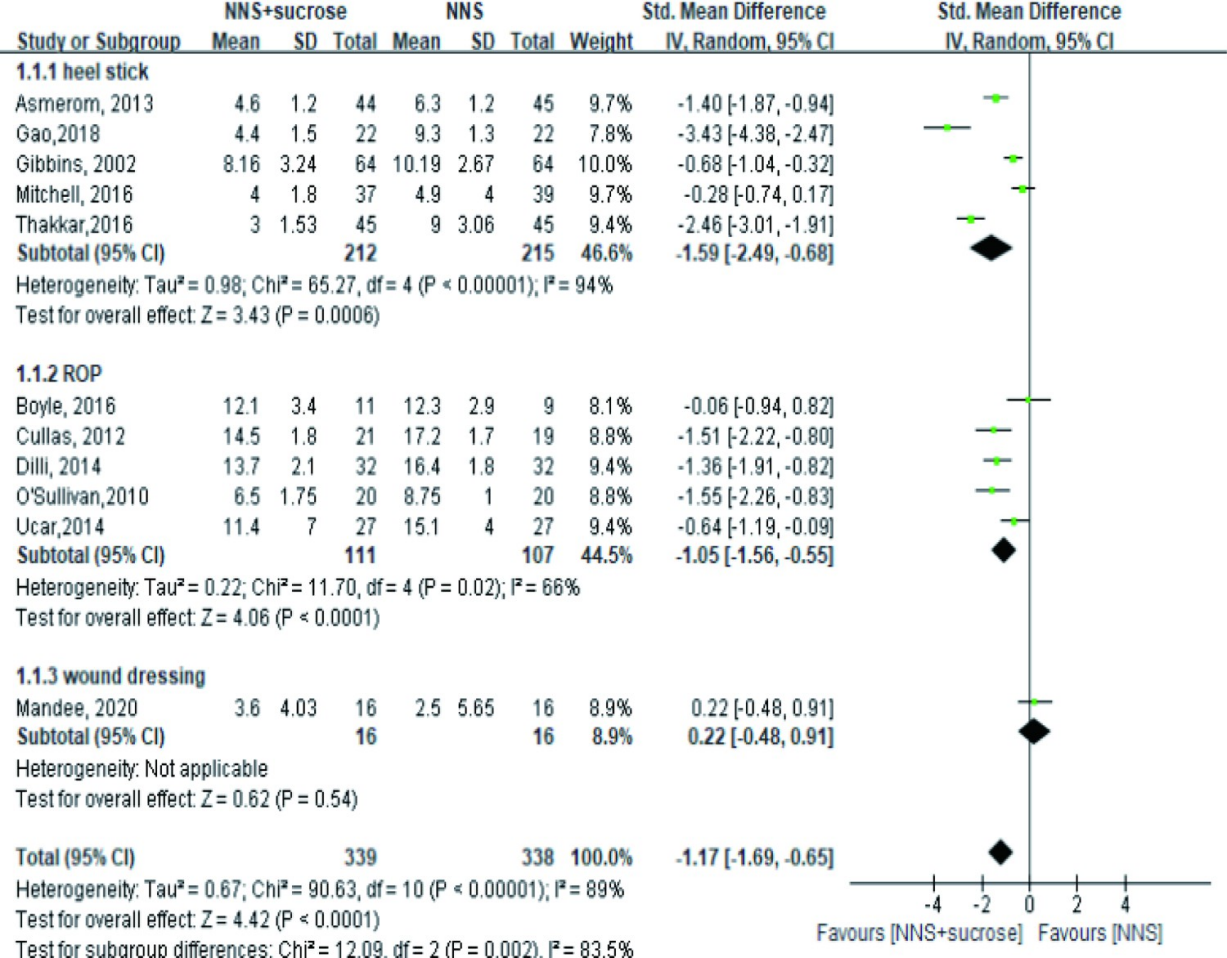
Forest plot of pain score comparing the combined intervention (NNS and sucrose) with applying NNS alone during different painful procedures. NNS: nonnutritive sucking.

A total of 11 studies with 677 participants explored the effect of OS combined with NNS compared with NNS alone during the painful procedures, most of which reported PIPP score, while one study reported NIPS [37] and one reported N-pass score [38]. In both heel-stick and ROP subgroups, a significant effect was observed under the random-effects model, with the standard mean difference being -1.59 (95%CI: -2.49, -0.68) and -1.05(95%CI: -1.56, -0.55), respectively. However, there was no significant difference in the wound dressing subgroup (Fig 3). A sensitivity analysis was conducted based on two aspects: removing conference abstracts and removing articles using non-PIPP measurements. The results remained significant after two conference abstracts were removed in the ROP group [33, 35]. Besides, unifying the measurements by removing two studies reporting NIPS or N-pass score did not change the results or reduce heterogeneity neither. The analysis showed no effect in four studies involving term infants regardless of the type of procedures (p = 0.16) with SMD: -0.81 (95%CI: -1.94, 0.33) [37, 39–41].

**Fig 4:**
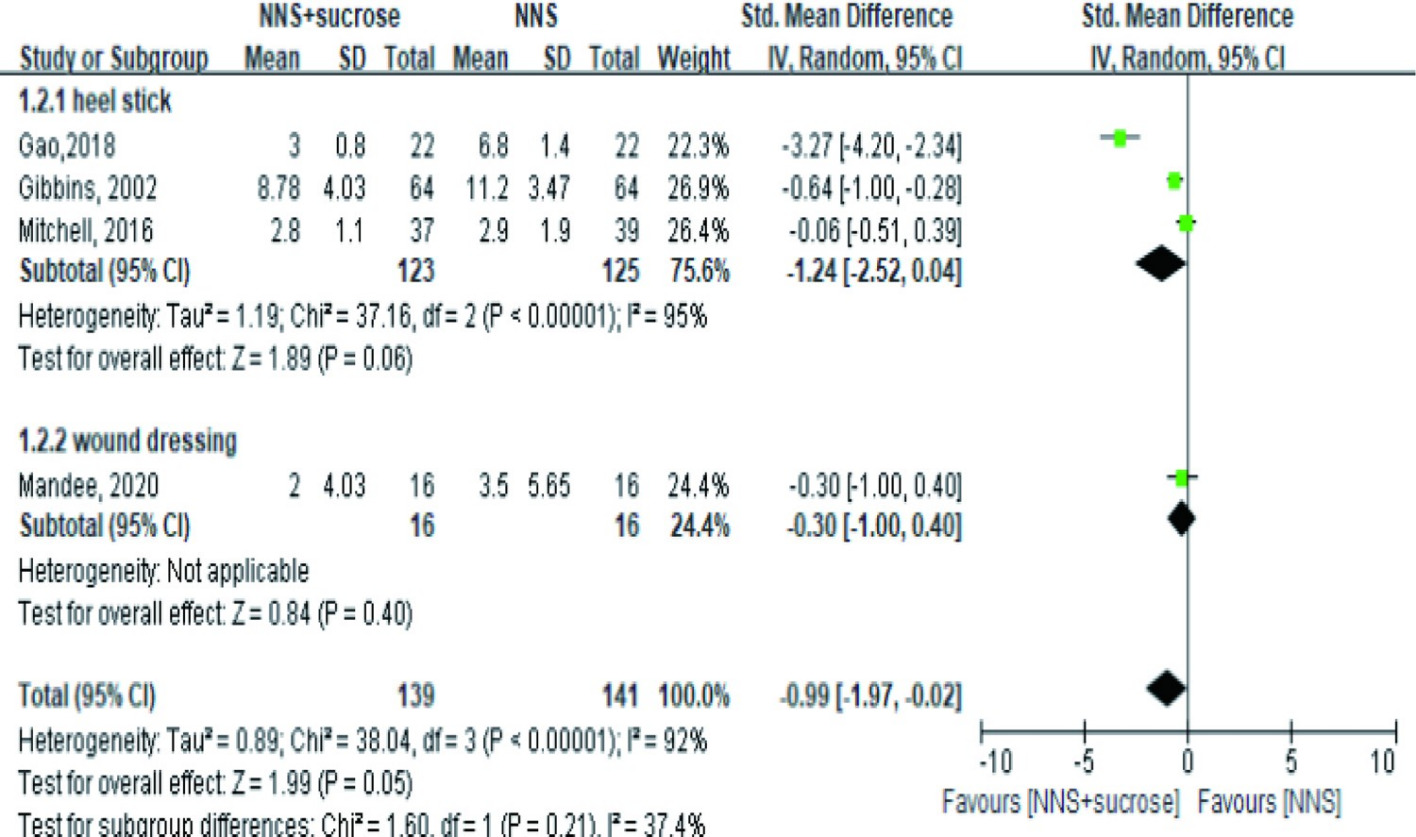
Forest plot of pain score comparing the combined intervention (NNS and sucrose) with applying NNS alone in the recovery phase. NNS: nonnutritive sucking.

Fewer studies provided information in the recovery phase. The available 4 studies showed the combined sucrose and NNS did not provide a better effect in relieving pain in both heel stick and wound dressing groups (Fig 4). However, this finding was not robust in the heel stick subgroup due to the reverse result (MD = -3.23, 95%CI: -4.56, -1.89, P<0.001) after removing Mitchell’s study in the sensitivity analysis [39].

#### 3.2.2. NNS+OS versus OS alone

**Fig 5:**
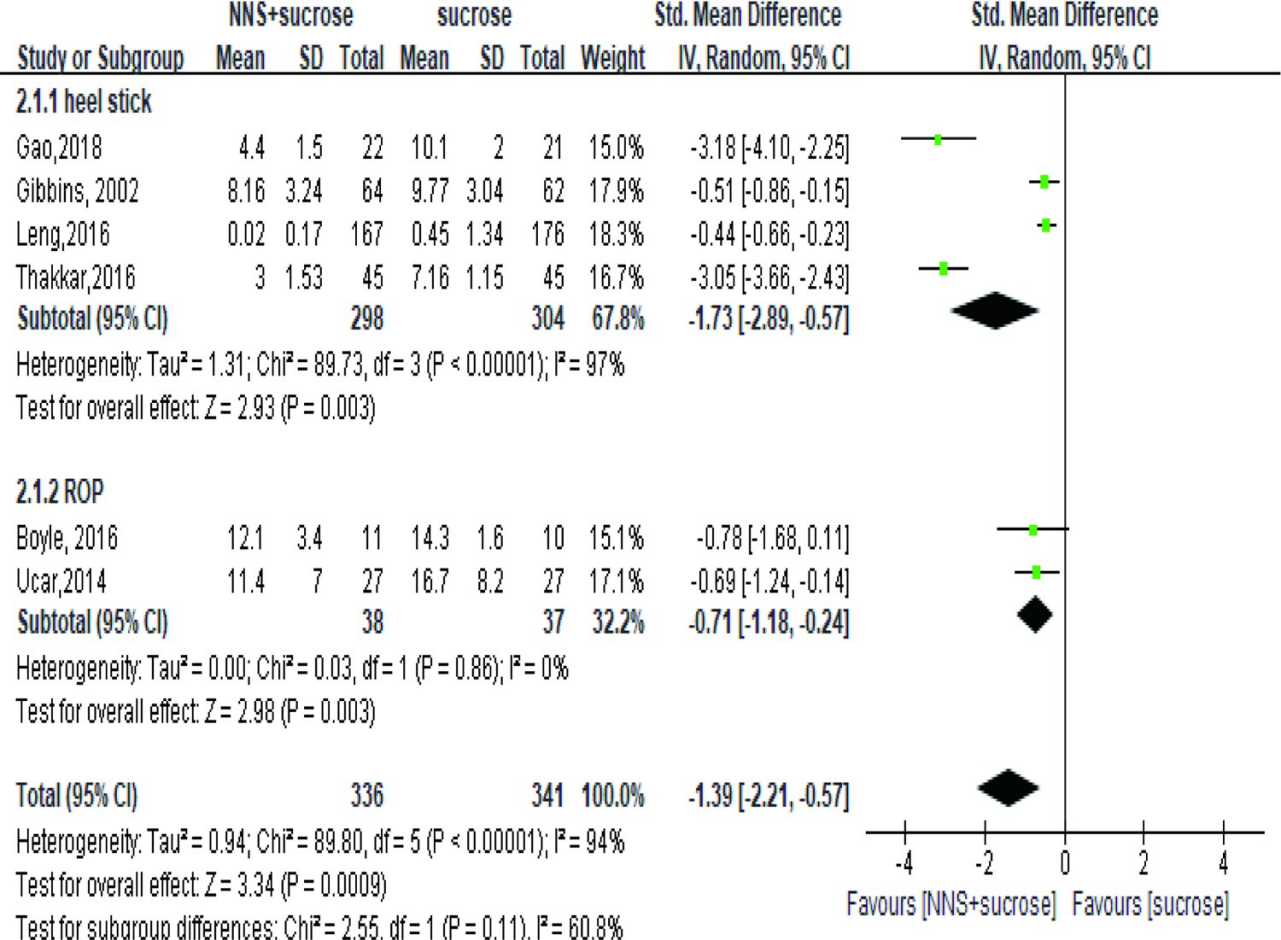
Forest plot of pain score comparing the combined intervention (NNS and sucrose) with applying sucrose alone during heel stick and ROP. NNS: nonnutritive sucking; ROP: retinopathy of prematurity.

**Fig 6:**
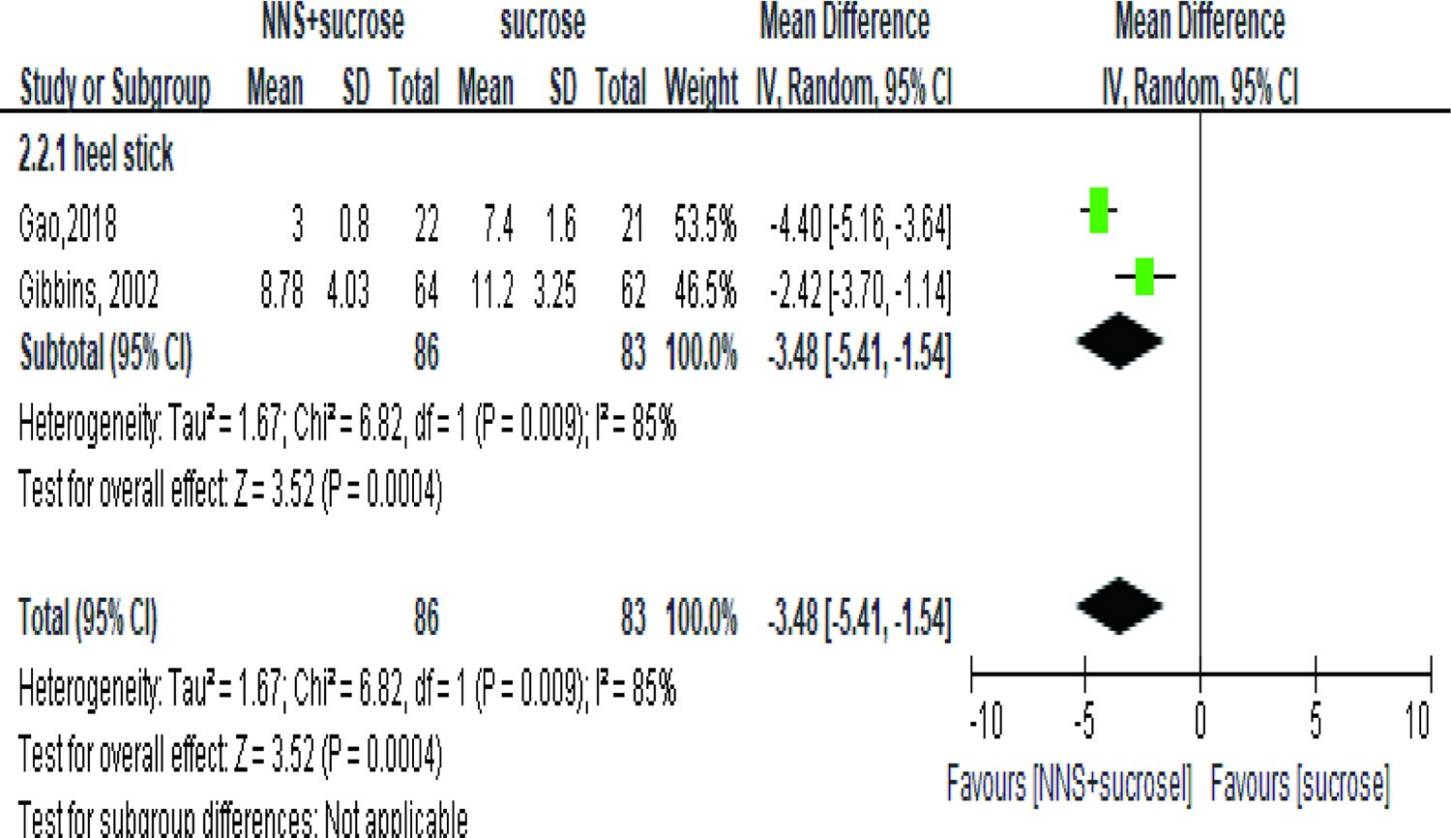
Forest plot of pain score comparing the combined intervention (NNS and sucrose) with applying sucrose alone in recovery phase. NNS: nonnutritive sucking.

Six studies [35, 40, 42–45] involving 677 infants or newborns assessed the pain in the group applying sucrose alone. One study measured pain as NFCS score [43] while the other five studies reported PIPP. The meta-analysis showed a significant effect of the combined interventions than OS (Fig 5). No significant difference, however, was detected when studying the term subgroup (SMD = -1.30, 95%CI: -2.81, 0.22, P = 0.09) [40, 41, 43]. In the recovery phase, the effect was also significant (MD = -3.48, 95%CI: -5.41, -1.54) (Fig 6).

#### 3.2.3 NNS+OS versus routine care group

**Fig 7:**
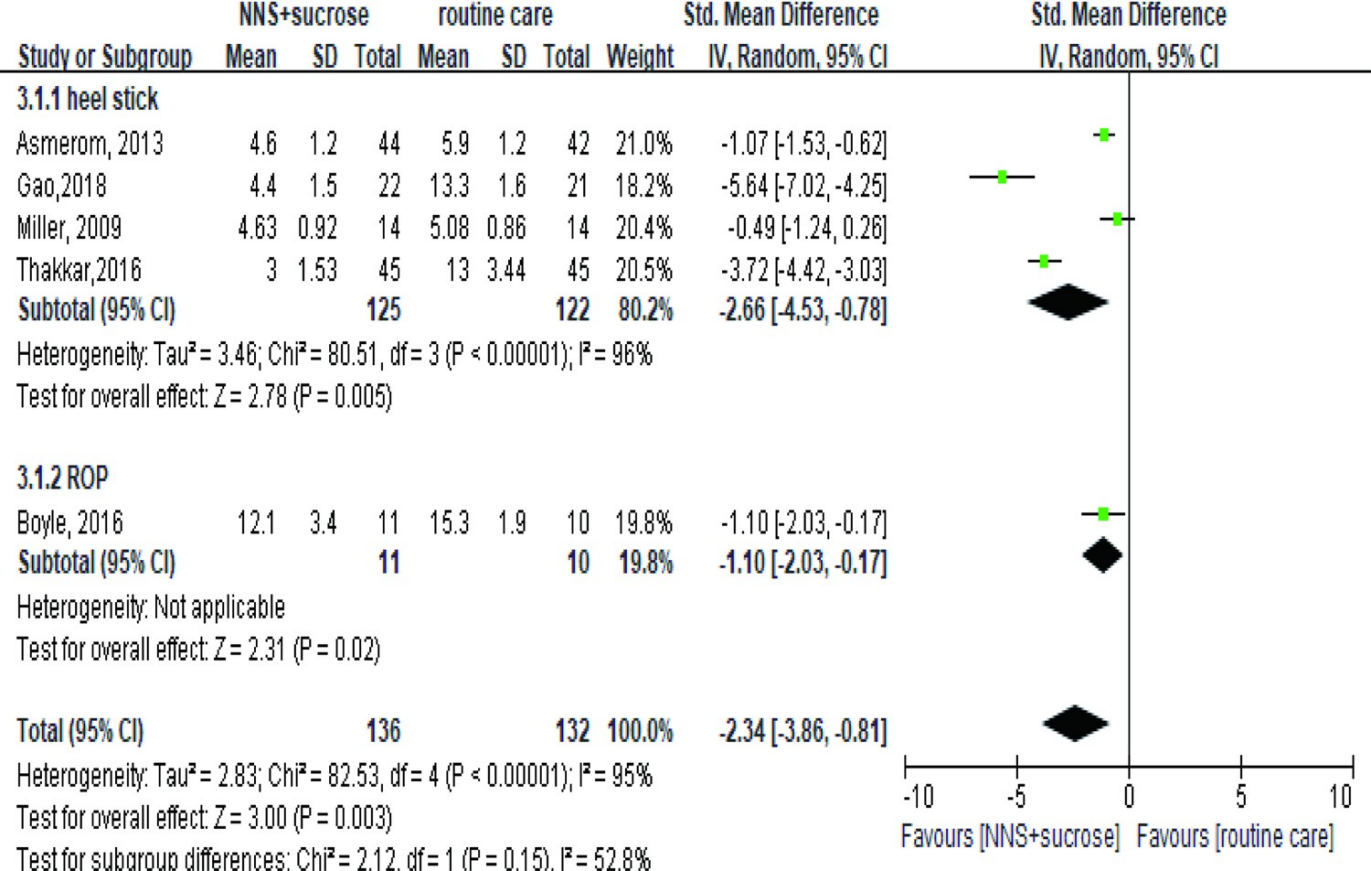
Forest plot of pain score comparing the combined intervention (NNS and sucrose) with routine care group during the procedures. NNS: nonnutritive sucking.

Five studies involving 268 participants were included in the analysis to compare the effect between the NNS+sucrose group and the routine care group [34, 40, 42, 44, 46]. The results showed a better impact of NNS+sucrose in both heel stick (SMD = -2.66, 95%CI: -4.53, -0.78) and ROP group (SMD = -1.1, 95%CI: -2.03,-0.17) (Fig 7). Removal of any study did not change the results significantly.

#### 3.2.4 NNS+OS versus breastfeeding or breast milk

**Fig 8:**
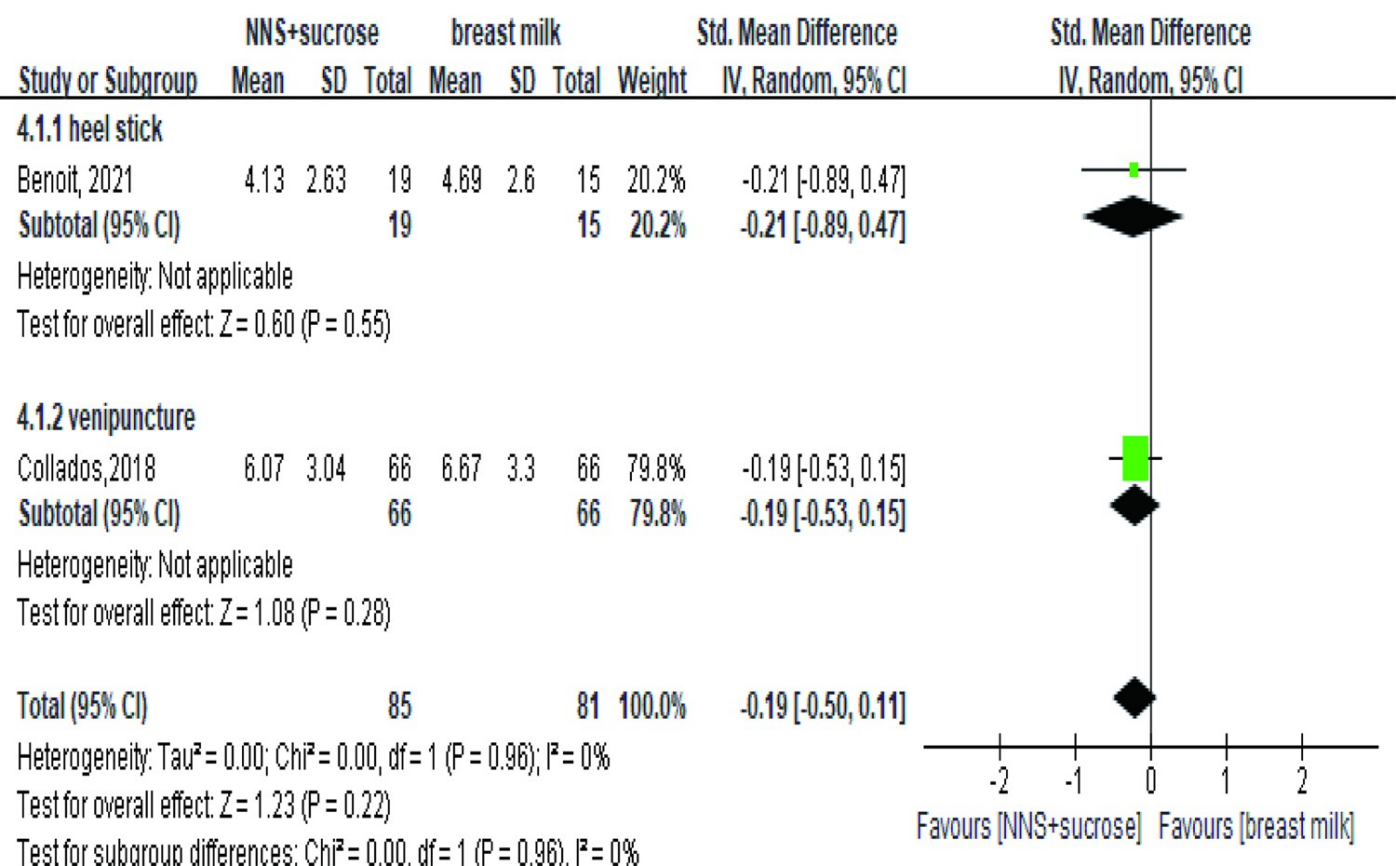
Forest plot of pain score comparing the combined intervention (NNS and sucrose) with breastmilk during the procedures. NNS: nonnutritive sucking.

The comparison between OS+NNS and breast milk showed no difference in effects for pain relief despite the heterogeneity in study design, intervention methods, pain procedures, outcome assessment and target population between the two studies (Fig 8). In Benoit’s study [47], full term infants underwent heel stick and were direct breastfed, whose pain was assessed by PIPP-R. In contrast, 66 preterm infants underwent venipuncture in two sequences. During the controlling period, infants were given breast milk via a pacifier, whose pain was assessed by PIPP in Collados’s study [36].

#### 3.2.5 NNS+OS versus glucose

Only one study enrolling 66 term newborns set glucose+NNS as the control group [48]. The result demonstrated a significant difference (p < 0.05) in both two phases. NIPS scores were significantly lower in the NNS+sucrose group (range 1–2 and median 0) compared with the NNS+glucose group (range 5–7 and median 6) during venipuncture.

#### 3.2.6 Publication bias

**Fig 9:**
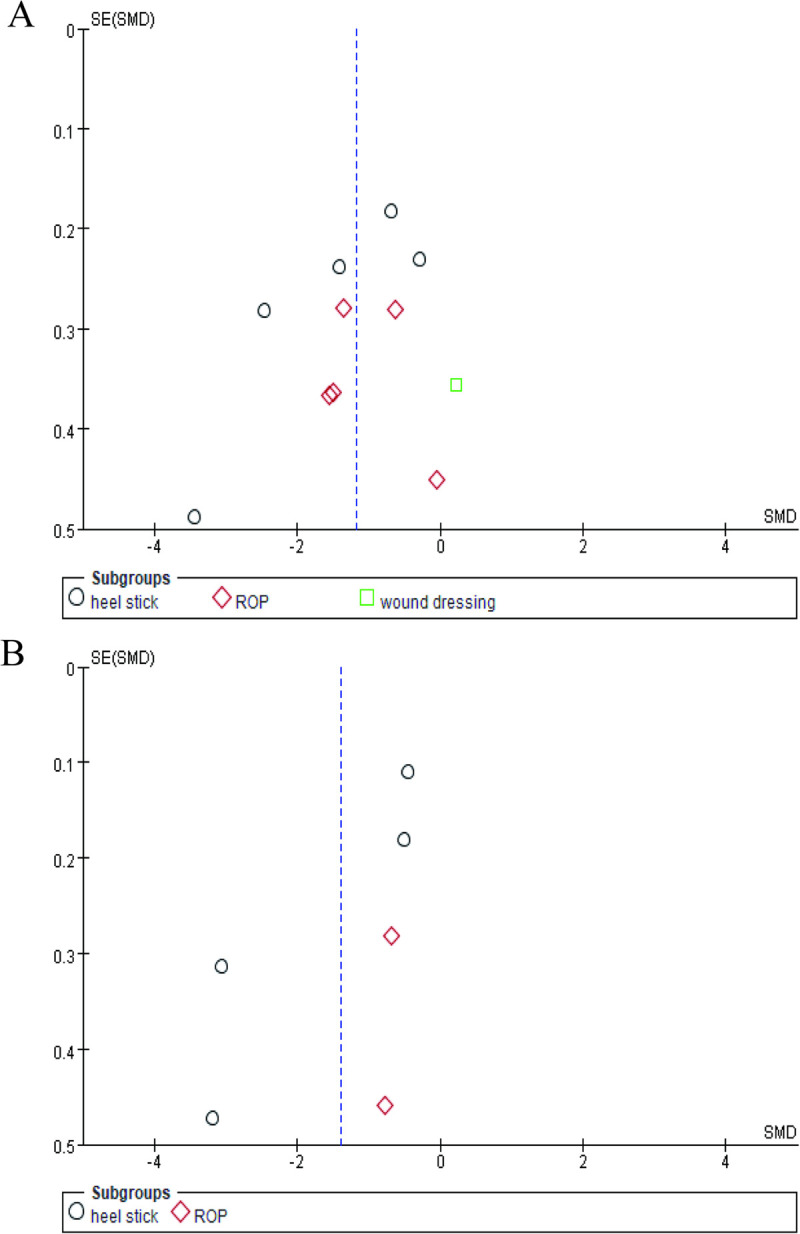
Funnel plots of publication bias on NNS+OS versus NNS alone (A) and NNS+OS versus OS alone (B) during painful procedures. NNS: nonnutritive sucking; OS: oral sucrose.

Publication bias was tested by Egger’s Test and funnel plots (Fig 9A, 9B). The results of Egger’s test indicated no publication bias in both scenarios. The bias (intercept) was estimated -3.3 (95%CI -10.90, 4.30) with p value 0.3515 in the comparison between NNS+OS and NNS alone (11 studies). The estimates of bias were -5.60 (95%CI -13.40, 2.20) with p value 0.117 in the comparison between NNS+OS and OS alone (6 studies). The funnel plots presented slight asymmetry especially in heel-stick subgroup. More studies are needed to justify this publication bias.

### 3.3 Adverse events

Among the included studies, ten trials [36–38, 40, 43–45, 47–49] reported the occurrence of adverse events. Five trials observed that the occurrence rate of adverse events was zero in all treatment groups [36, 37, 43, 47, 48]. Oxygen desaturation was the most common adverse event, reported in 4 trials [38, 40, 45, 49]. Gao reported 5 episodes of vomit and abdominal distension whereas Dilli and O’Sullivan reported 13 and 4 episodes of bradycardia, respectively. A significant difference in tachycardia between the NNS+sucrose and the NNS alone group was observed by Dilli, whereas no significant adverse effects were observed in other trials (Table 2). Three trials reported heart rate and oxygen saturation as mean (SD) as a secondary outcome (Table 3) [34, 44, 46]. They all observed a significant effect of NNS+sucrose on oxygen saturation and heart rate except a non-significant result in Asmerom’s for heart rate.

**Table 2:** Episodes of adverse events in included studies.

Study	NNS+sucrose: N(Total)	NNS alone: N(Total)	Sucrose alone: N (Total)	Routine care: N(Total)	Clinical significance
Dilli, 2014 [49]	bradycardia:6(32)	bradycardia:7 (32)	-	-	bradycardia: p = 0.75
	tachycardia:12(32)	tachycardia:19 (32)			tachycardia: p = 0.08
	desaturations:6(32)	desaturations:7 (32)			desaturations: p = 0.75
Gao, 2018 [44]	vomit:1(22)	vomit:1(22)	-	Vomit:1	vomit: *p* = 0.800;
				abdominal distension:1	abdominal distension: *p* = 0.562
	abdominal distension:1(22)				
Gibbins, 2002 [45]	No adverse events	oxygen desaturation:2(64)	oxygen desaturation:3(64)	-	p>0.05
		choke:1(64)			
O’Sullivan, 2010 [38]	bradycardia:1(20)	bradycardia:3(20)	-	-	bradycardia: p = 0.328
	desaturations:1(20)	desaturations:3(20)			desaturations: p = 0.328
Thakkar, 2016 [40]	oxygen desaturation:1(45)	oxygen desaturation:1(45)	oxygen desaturation:1(45)	oxygen desaturation:2(45)	p>0.05

**Table 3:** Summarized statistics of oxygen desaturation and Heart rate in included studies during painful procedures.

Study	Sucrose+NNS		NNS		sucrose		Routine care		p-value
	Mean (SD)	N	Mean (SD)	N	Mean (SD)	N	Mean (SD)	N	
oxygen saturation									
Asmerom, 2013 [46]	96.4(0.6)	44	95.8(0.6)	45	-	-	-	-	<0.0001
Gao, 2018 [44]	95.2(1.6)	22	92.9(2.4)	22	93.5(1.7)	21	92.9(2.1)	21	<0.05
Miller, 2009 [34]	97.69(2.41)	14	-	-	-	-	94.30(3.74)	14	<0.001
Heart rate									
Asmerom, 2013 [46]	170.5(14.7)	44	164.9(14.6)	45	-	-	-	-	0.07
Gao, 2018 [44]	138.6(7.9)	22	154.2(9.0)	22	151.6(9.6)	21	156.8(7.2)	21	<0.0001
Miller, 2009 [34]	135.64(7.71)	14	-	-	-	-	150.64(7.53)	14	<0.001

NNS: nonnutritive sucking; SD: Standard deviation.

## 4 Discussion

Pain management has been well established in recent years, especially in procedures involving skin punctures [12, 50]. However, there is not a practical guideline on how to reduce pain and stress in a prolonged procedure such as ROP and wound treatment [51]. Therefore, this systematic review and meta-analysis aims to determine the effect of combined NNS and sucrose intervention in different types of painful procedures.

Overall, the combined interventions of NNS and sucrose showed the superiority in relieving mild pain during the painful procedures compared to applying NNS alone or sucrose alone or standard care. This has been confirmed by previous reviews [10, 13]. Non-nutritive sucking was believed to be associated with antinociceptive mechanisms and sucrose appears to enhance the effect of NNS, leading to an increase of endogenous endorphins [9, 52]. However, the mechanism of the combined effects is not completely clear [53].

On the other hand, the superiority is not significant when compared with direct breastfeeding or giving breast milk via a pacifier [36, 47]. The similarity between sucking through a pacifier and breastfeeding might explain this result [52]. Besides, swaddling and a blanket used in the two trials helped establish a simulated environment as breastfeeding, calming and comforting the babies. The similar effectiveness in healthy term neonates or stable late preterm neonates between sucrose/glucose administration and breast milk was also proposed in Shah’s review [54].

ROP examinations and wound dressing have two points in common. Both procedures last for several minutes and tend to give giving stronger painful stimuli. The insertion of an eyelid speculum and scleral depression usually increases the intensity of the pain during ROP screening [55]. Although a significant effect was observed in ROP examinations in a preterm population, we found the means of PIPP score even in the combined NNS and sucrose group still around or higher than 12, qualitatively, indicating a moderate-to-severe level. Considering the preterm infants tend to have lower scores than term infants, such high scores indicated the finite effect of this combined intervention [56]. Besides, a non-significant effect was detected during wound dressing in Mandee’s study where moderate-to-severe pain were often observed [37]. This might not be indicative because few studies researched on pain managements in wound dressing. However, previous studies found the effect of sucrose were time-dependent and could not last for a long time [10, 41]. This might be able to explain the minor effect. More innovations are needed on non-pharmacological interventions for those intensive procedures. For example, a recent study recommended the physiological flexion position called ROP position and it was demonstrated more effective than the combination of sucrose and NNS [57]. The combination of multiple non-pharmacological methods, including non-nutritive sucking, oral breast milk, and facilitated tucking, music, etc. might also help enhance the effects of pain relief [17, 18, 58]. Investigations on more effective methods are warranted, especially for lowering moderate or even severe pain.

Few studies in our analysis reported the pain measurements in the recovery phase, which might lead to an unreliable result in the meta-analysis. Our analysis found the effect has vanished in heel stick procedure when comparing the combined interventions with NNS alone group during this period. This finding could be reversed by removing a study with nonsignificant result in sensitivity analysis [39]. In this trial, both intervention and control groups received a pacifier and facilitated tucking resulting a low PIPP and sucrose did not contribute much to reducing pain. In fact, facilitated tucking has been reported to be effective in pain management [59].

Subgroup analysis on sucrose administration was not performed in the meta-analysis because of the varieties in different trials in terms of volumes (from 0.1 mL to 2 mL), concentrations (range from 12% to 33%) and frequencies. However, Steven found no difference in effectiveness among doses by proposing the minimal effective dose of only 0.1 mL in relieving procedural pain [60]. Thus, the wide range of volumes of sucrose might not influence the comparison.

OS has been considered to cause adverse events such as desaturation and bradycardia, especially in preterm infants [10]. Our analysis indeed indicates the trend of oxygen saturation increasing while heart rate decreasing under intervention. However, we did not observe a higher occurrence of bradycardia, tachycardia or desaturations in groups with OS. Besides, adverse events indeed occurred more in preterm subjects. Trials with zero adverse events were mostly based on full-term infants. This might indicate the vulnerability of preterm infants attributes more to adverse events than OS. Nonetheless, the evidence might be influenced by reporting bias because some studies did not report any information on the safety during the intervention procedure.

Although the combined intervention of sucrose and NNS was considered effective in reducing pain among the studies included, sucrose was often administered 2 min prior to the painful procedure in these studies. This time interval was unjustified by Meesters in 2017 [61]. Besides, the recovery phase was neglected in half of the studies. The recovery from pain is important as it also affects the baby in a long-term way. We might improve the intervention methods to deal with moderate and severe pain and pay more attention to the recovery phase.

De Bernardo’s trial found sucrose was more effective then oral glucose when both combined with a pacifier. This conflicts with a previous review which take glucose as an acceptable alternative to sucrose [62]. More studies could be conducted in this direction in order to expand the available and effective intervention methods.

There are three limitations in our analysis. Firstly, the heterogeneity is non-negligible in heel-stick subgroups but acceptable in other painful procedures. Heel-stick was involved in most studies. The variations in their protocols, such as population, the operating process, the pain measurements, or the sample size might interactively contribute to moderate to high heterogeneity. For example, some studies enrolled both preterm and term infants in the trial, while others enrolled purely preterm or full-term infants, which added the difficulty in explaining the effects on the certain population. Different scales and standards in pain measurements also influenced pooling data in quantitative analysis. We have conducted post hoc subgroup analyses to test the source of heterogeneity in terms of these factors. High heterogeneity might induce an unreliable conclusion even under a random-effects model. Results should be explained with caution. Secondly, pain score was the only one outcome to measure pain in this analysis and the different assessments were pooled. Those pain assessment methods are distinguishable from their target population, scales and evaluation items [63]. It was hard to unify because PIPP was the most common and other assessments accounted for no more than 25% of included studies. A sensitivity analysis was conducted to reduce the potential bias though. Other measurements including crying time were not investigated because of the limited number of studies. There were very few studies reporting crying time and comparing the combined intervention with sucrose alone or routine care. A single outcome of pain score might not be robust enough since different scales were used among studies. Finally, publication bias seems to exist in our analysis.

## 5 Conclusion

In conclusion, our systematic review and meta-analysis indicated the superiority of the combined intervention of sucrose and NNS than any single intervention except for breastfeeding. However, the effect appears to be mild in alleviating moderate-to-severe pain. More exploration and improvement of intervention were needed.

## Supporting information

S1 Checklist PRISMA 2009 checklist. (DOCX)

S1 Table Full searching strategy (from Jan 1, 2000 to Mar 31, 2021). (DOCX)

## Abbreviations

NICU: neonatal intensive care unit
OS: oral sucrose
NNS: nonnutritive sucking
PIPP: Premature Infant Pain Profile
PIPP: R: premature infant pain profile-revised
NIPS: Neonatal Infant Pain Scale
NFCS: Neonatal Facial Coding System
N-PASS: Neonatal Pain Agitation and Sedation Scale
WMD: weighted mean difference
SMD: standardized mean difference
ROP: retinopathy of prematurity
SDs: standard deviations

## References

[1] Carbajal R, Rousset A, Danan C, Coquery S, Nolent P, Ducrocq S, et al. Epidemiology and treatment of painful procedures in neonates in intensive care units. JAMA. 2008;300(1):60–70. doi: 10.1001/jama.300.1.60 . 18594041

[2] Squillaro A, Mahdi EM, Tran N, Lakshmanan A, Kim E, Kelley-Quon LI. Managing Procedural Pain in the Neonate Using an Opioid-sparing Approach. Clin Ther. 2019;41(9):1701–13. doi: 10.1016/j.clinthera.2019.07.014 . 31431300 PMC6790974

[3] Bouza H. The impact of pain in the immature brain. J Matern Fetal Neonatal Med. 2009;22(9):722–32. doi: 10.3109/14767050902926962 . 19526425

[4] Doesburg SM, Chau CM, Cheung TPL, Moiseev A, Ribary U, Herdman AT, et al. Neonatal pain-related stress, functional cortical activity and visual-perceptual abilities in school-age children born at extremely low gestational age. Pain. 2013;154(10):1946–52. doi: 10.1016/j.pain.2013.04.009 . 23711638 PMC3778166

[5] Jeong IS, Park SM, Lee JM, Choi YJ, Lee J. Perceptions on pain management among Korean nurses in neonatal intensive care units. Asian Nurs Res. 2014;8(4):261–6. doi: 10.1016/j.anr.2014.05.008 . 25529908

[6] Kyololo OM, Stevens B, Gastaldo D, Gisore P. Procedural pain in neonatal units in Kenya. Arch Dis Child Fetal Neonatal Ed 2014;99(6):F464–7. doi: 10.1136/archdischild-2014-306003 . 24996597

[7] Johnston C, Barrington KJ, Taddio A, Carbajal R, Filion F. Pain in Canadian NICUs: have we improved over the past 12 years? Clin J Pain. 2011;27(3):225–32. doi: 10.1097/AJP.0b013e3181fe14cf . 21178602

[8] Olsen EA, Brambrink AM. Anesthetic neurotoxicity in the newborn and infant. Curr Opin Anaesthesiol. 2013;26(5):535–42. doi: 10.1097/01.aco.0000433061.59939.b7 . 23995065

[9] Lim Y, Godambe S. Prevention and management of procedural pain in the neonate: an update, American Academy of Pediatrics, 2016. Arch Dis Child Educ Pract Ed. 2017;102(5):254–6. doi: 10.1136/archdischild-2016-311066 . 28724533

[10] Stevens B, Yamada J, Ohlsson A, Haliburton S, Shorkey A. Sucrose for analgesia in newborn infants undergoing painful procedures. Cochrane Database Syst Rev. 2016;7(7):Cd001069. doi: 10.1002/14651858.CD001069.pub5 . 27420164 PMC6457867

[11] Zeller B, Giebe J. Pain in the neonate: focus on nonpharmacologic interventions. Neonatal Netw. 2014;33(6):336–40. doi: 10.1891/0730-0832.33.6.336 . 25391593

[12] McNair C, Campbell-Yeo M, Johnston C, Taddio A. Nonpharmacologic Management of Pain During Common Needle Puncture Procedures in Infants: Current Research Evidence and Practical Considerations: An Update. Clin Perinatol. 2019;46(4):709–30. doi: 10.1016/j.clp.2019.08.006 . 31653304

[13] Liu Y, Huang X, Luo B, Peng W. Effects of combined oral sucrose and nonnutritive sucking (NNS) on procedural pain of NICU newborns, 2001 to 2016: A PRISMA-compliant systematic review and meta-analysis. Medicine (Baltimore). 2017;96(6):e6108. doi: 10.1097/MD.0000000000006108 . 28178172 PMC5313029

[14] Hatfield LA, Murphy N, Karp K, Polomano RC. A Systematic Review of Behavioral and Environmental Interventions for Procedural Pain Management in Preterm Infants. J Pediatr Nurs. 2019;44:22–30. doi: 10.1016/j.pedn.2018.10.004 . 30683278

[15] Thrane SE, Wanless S, Cohen SM, Danford CA. The Assessment and Non-Pharmacologic Treatment of Procedural Pain From Infancy to School Age Through a Developmental Lens: A Synthesis of Evidence With Recommendations. J Pediatr Nurs. 2016;31(1):e23–32. doi: 10.1016/j.pedn.2015.09.002 . 26424196 PMC4724566

[16] Shah V, Taddio A, McMurtry CM, Halperin SA, Noel M, Pillai Riddell R, et al. Pharmacological and Combined Interventions to Reduce Vaccine Injection Pain in Children and Adults: Systematic Review and Meta-Analysis. Clin J Pain. 2015;31(10 Suppl):S38–63. doi: 10.1097/AJP.0000000000000281 . 26201016 PMC4900424

[17] Qiu J, Jiang YF, Li F, Tong QH, Rong H, Cheng R. Effect of combined music and touch intervention on pain response and β-endorphin and cortisol concentrations in late preterm infants. BMC Pediatr. 2017;17(1):38. doi: 10.1186/s12887-016-0755-y . 28125980 PMC5270209

[18] Peng HF, Yin T, Yang L, Wang C, Chang YC, Jeng MJ, et al. Non-nutritive sucking, oral breast milk, and facilitated tucking relieve preterm infant pain during heel-stick procedures: A prospective, randomized controlled trial. Int J Nurs Stud. 2018;77:162–70. doi: 10.1016/j.ijnurstu.2017.10.001 . 29100198

[19] Shah SR, Kadage S, Sinn J. Trial of Music, Sucrose, and Combination Therapy for Pain Relief during Heel Prick Procedures in Neonates. J Pediatr. 2017;190:153–8.e2. doi: 10.1016/j.jpeds.2017.08.003 . 29144240

[20] Elserafy FA, Alsaedi SA, Louwrens J, Bin Sadiq B, Mersal AY. Oral sucrose and a pacifier for pain relief during simple procedures in preterm infants: a randomized controlled trial. Ann Saudi Med. 2009;29(3):184–8. doi: 10.4103/0256-4947.52821 . 19448377 PMC2813645

[21] Liaw JJ, Zeng WP, Yang L, Yuh YS, Yin T, Yang MH. Nonnutritive sucking and oral sucrose relieve neonatal pain during intramuscular injection of hepatitis vaccine. J Pain Symptom Manage. 2011;42(6):918–30. doi: 10.1016/j.jpainsymman.2011.02.016 . 21620644

[22] Higgins JP GS. Cochrane Handbook for Systematic Reviews of Interventions Version 5.1.0. The Cochrane Collaboration. 2011. https://www.cochrane-handbook.org/.

[23] Stevens B, Johnston C, Petryshen P, Taddio A. Premature Infant Pain Profile: development and initial validation. Clin J Pain. 1996;12(1):13–22. doi: 10.1097/00002508-199603000-00004 . 8722730

[24] Stevens BJ, Gibbins S, Yamada J, Dionne K, Lee G, Johnston C, et al. The premature infant pain profile-revised (PIPP-R): initial validation and feasibility. Clin J Pain. 2014;30(3):238–43. doi: 10.1097/AJP.0b013e3182906aed . 24503979

[25] Lawrence J, Alcock D, McGrath P, Kay J, MacMurray SB, Dulberg C. The development of a tool to assess neonatal pain. Neonatal Netw. 1993;12(6):59–66. doi: 10.1016/0885-3924(91)91127-U 8413140

[26] Peters JW, Koot HM, Grunau RE, de Boer J, van Druenen MJ, Tibboel D, et al. Neonatal Facial Coding System for assessing postoperative pain in infants: item reduction is valid and feasible. Clin J Pain. 2003;19(6):353–63. doi: 10.1097/00002508-200311000-00003 . 14600535

[27] Hummel P, Lawlor-Klean P, Weiss MG. Validity and reliability of the N-PASS assessment tool with acute pain. J Perinatol. 2010;30(7):474–8. doi: 10.1038/jp.2009.185 . 19924132

[28] Wan X, Wang W, Liu J, Tong T. Estimating the sample mean and standard deviation from the sample size, median, range and/or interquartile range. BMC Med Res Methodol. 2014;14:135. doi: 10.1186/1471-2288-14-135 . 25524443 PMC4383202

[29] Pudar-Hozo S, Djulbegovic B, Hozo I. Estimating the mean and variance from the median, range, and the size of a sample. BMC Med Res Methodol. 2005;13:1–10. doi: 10.1186/1471-2288-5-13 . 15840177 PMC1097734

[30] Bland M. Estimating Mean and Standard Deviation from the Sample Size, Three Quartiles, Minimum, and Maximum. Int J Stat Med Res. 2015;4:57–64. doi: 10.6000/1929-6029.2015.04.01.6 .25524443

[31] Elbourne DR, Altman DG, Higgins JP, Curtin F, Worthington HV, Vail A. Meta-analyses involving cross-over trials: methodological issues. Int J Epidemiol. 2002;31(1):140–9. doi: 10.1093/ije/31.1.140 . 11914310

[32] The Nordic Cochrane Centre TCC. Review Manager (RevMan). Version 5.3. Copenhagen: The Nordic Cochrane Centre, The Cochrane Collaboration. 2014.

[33] Cullas Ilarslan N, Dilli D, Kabatas EU, Beken S, Aydin B, Zencirotlu A, et al. Predicting the efficacy of oral sucrose in reducing pain during ophtalmological examination for retinopathy of prematurity: A prospective randomised study. Arch Dis Child. 2012;97(Suppl 2):A55–A55. 10.1136/archdischild-2012-302724.0190.

[34] Miller HD. Nonnutritive sucking and sucrose-induced analgesia: effect on heart rate, oxygen saturation, and pain in intubated infants. Adv Neonatal Care. 2009:142 p. doi: 10.1097/01.ANC.0000360173.97702.0d

[35] Ucar S, Varma M, Altan S. The efficacy of non-nutritive sucking and sucrose for the relief of pain during eye examinations for retinopathy of prematurity: A randomised controlled trial. Arch Dis Child. 2014;2):A182–A3. doi: 10.1136/archdischild-2014-307384.491

[36] Collados-Gómez L, Ferrera-Camacho P, Fernandez-Serrano E, Camacho-Vicente V, Flores-Herrero C, García-Pozo AM, et al. Randomised crossover trial showed that using breast milk or sucrose provided the same analgesic effect in preterm infants of at least 28 weeks. Acta Paediatr. 2018;107(3):436–41. doi: 10.1111/apa.14151 . 29150862

[37] Mandee S, Buachai K, Aroonpruksakul N, Tantemsapya N, Buasuk T,. Effects of Sucrose and Nonnutritive Sucking on Pain Behavior in Neonates and Infants undergoing Wound Dressing after Surgery: A Randomized Controlled Trial. Eur J Pediatr. 2020. doi: 10.1055/s-0040-1716883 . 32942327

[38] O’Sullivan A, O’Connor M, Brosnahan D, McCreery K, Dempsey EM. Sweeten, soother and swaddle for retinopathy of prematurity screening: a randomised placebo controlled trial. Arch Dis Child Fetal Neonatal Ed. 2010;95(6):F419–22. doi: 10.1136/adc.2009.180943 . 20876596

[39] Mitchell AJ, Hall RW, Golianu B, Yates C, Williams DK, Chang J, et al. Does noninvasive electrical stimulation of acupuncture points reduce heelstick pain in neonates? Acta Paediatr. 2016;105(12):1434–9. doi: 10.1111/apa.13581 . 27607517 PMC5118154

[40] Thakkar P, Arora K, Goyal K, Das R. To evaluate and compare the efficacy of combined sucrose and non-nutritive sucking for analgesia in Newborns undergoing minor painful procedure: A randomised controlled trial. J Perinatol. 2016 Jan;36(1):67–70. doi: 10.1038/jp.2015.122 . 26583940

[41] Gibbins S, Stevens B. The influence of gestational age on the efficacy and short-term safety of sucrose for procedural pain relief. Adv Neonatal Care. 2003;3(5):241–9. . 14648521

[42] Boyle EM, Freer Y, Khan-Orakzai Z, Watkinson M, Wright E, Ainsworth JR, et al. Sucrose and non-nutritive sucking for the relief of pain in screening for retinopathy of prematurity: a randomised controlled trial. Arch Dis Child Fetal Neonatal Ed. 2006;91(3):F166–8. doi: 10.1136/adc.2005.087668 . 16428355 PMC2672697

[43] Leng HY, Zheng XL, Zhang XH, He HY, Tu GF, Fu Q, et al. Combined non-pharmacological interventions for newborn pain relief in two degrees of pain procedures: A randomized clinical trial. Eur J Pain. 2016;20(6):989–97. doi: 10.1002/ejp.824 . 26685099

[44] Gao H, Li M, Gao H, Xu G, Li F, Zhou J, et al. Effect of non-nutritive sucking and sucrose alone and in combination for repeated procedural pain in preterm infants: A randomized controlled trial. Int J Nurs Stud. 2018;83:25–33. doi: 10.1016/j.ijnurstu.2018.04.006 . 29684832

[45] Gibbins S, Stevens B, Hodnett E, Pinelli J, Ohlsson A, Darlington G. Efficacy and safety of sucrose for procedural pain relief in preterm and term neonates. Nurs Res. 2002;51(6):375–82. doi: 10.1097/00006199-200211000-00005 . 12464757

[46] Asmerom Y, Slater L, Boskovic DS, Bahjri K, Holden MS, Phillips R, et al. Oral sucrose for heel lance increases adenosine triphosphate use and oxidative stress in preterm neonates. J Pediatr. 2013;163(1):29–35.e1. doi: 10.1016/j.jpeds.2012.12.088 . 23415615 PMC3687041

[47] Benoit B, Newman A, Martin-Misener R, Latimer M, Campbell-Yeo M. The influence of breastfeeding on cortical and bio-behavioural indicators of procedural pain in newborns: Findings of a randomized controlled trial. Early Hum Dev. 2021;154:105308. doi: 10.1016/j.earlhumdev.2021.105308 . 33513546

[48] De Bernardo G, Riccitelli M, Sordino D, Giordano M, Piccolo S, Buonocore G, et al. Oral 24% sucrose associated with nonnutritive sucking for pain control in healthy term newborns receiving venipuncture beyond the first week of life. J Pain Res. 2019;12:299–305. doi: 10.2147/JPR.S184504 . 30662283 PMC6329346

[49] Dilli D, İlarslan NE, Kabataş EU, Zenciroğlu A, Şimşek Y, Okumuş N. Oral sucrose and non-nutritive sucking goes some way to reducing pain during retinopathy of prematurity eye examinations. Acta Paediatr. 2014;103(2):e76–9. doi: 10.1111/apa.12454 . 24730361

[50] Hall RW, Anand KJS. Pain management in newborns. Clin Perinatol. 2014;41(4):895–924. doi: 10.1016/j.clp.2014.08.010 . 25459780 PMC4254489

[51] Pirelli A, Savant Levet P, Garetti E, Ancora G. Literature review informs clinical guidelines for pain management during screening and laser photocoagulation for retinopathy of prematurity. Acta Paediatr. 2019;108(4):593–9. doi: 10.1111/apa.14523 . 30054933

[52] Gibbins S, Stevens B. Mechanisms of sucrose and non-nutritive sucking in procedural pain management in infants. Pain Res Manag. 2001;6(1):21–8. doi: 10.1155/2001/376819 . 11854758

[53] de Freitas RL, Kübler JM, Elias-Filho DH, Coimbra NC. Antinociception induced by acute oral administration of sweet substance in young and adult rodents: the role of endogenous opioid peptides chemical mediators and μ(1)-opioid receptors. Pharmacol Biochem Behav. 2012;101(2):265–70. doi: 10.1016/j.pbb.2011.12.005 . 22197708

[54] Shah PS, Herbozo C, Aliwalas LL, Shah VS. Breastfeeding or breast milk for procedural pain in neonates. Cochrane Database Syst Rev. 2012(12). doi: 10.1002/14651858.CD004950.pub3 23235618 PMC10108374

[55] Grabska J, Walden P, Lerer T, Kelly C, Hussain N, Donovan T, et al. Can Oral Sucrose Reduce the Pain and Distress Associated with Screening for Retinopathy of Prematurity? J Perinatol. 2005;25(1):33–5. doi: 10.1038/sj.jp.7211199 . 15343351

[56] Ballantyne M, Stevens B, McAllister M, Dionne K, Jack A. Validation of the premature infant pain profile in the clinical setting. Clin J Pain. 1999;15(4):297–303. doi: 10.1097/00002508-199912000-00006 . 10617258

[57] Metreş Ö, Yıldız S. Pain Management with ROP Position in Turkish Preterm Infants During Eye Examinations: A Randomized Controlled Trial. J Pediatr Nurs. 2019;49:e81–e9. doi: 10.1016/j.pedn.2019.08.013 Epub 2019 Sep 5 . 31495577

[58] Uematsu H, Sobue I. Effect of music (Brahms lullaby) and non-nutritive sucking on heel lance in preterm infants: A randomized controlled crossover trial. Paediatr Child Health. 2019;24(1):e33–e9. doi: 10.1093/pch/pxy072 . 30792607 PMC6376306

[59] Gomes Neto M, da Silva Lopes IA, Araujo A, Oliveira LS, Saquetto MB. The effect of facilitated tucking position during painful procedure in pain management of preterm infants in neonatal intensive care unit: a systematic review and meta-analysis. Eur J Pediatr. 2020;179(5):699–709. doi: 10.1007/s00431-020-03640-5 . 32222816

[60] Stevens B, Yamada J, Campbell-Yeo M, Gibbins S, Harrison D, Dionne K, et al. The minimally effective dose of sucrose for procedural pain relief in neonates: a randomized controlled trial. BMC Pediatr. 2018;18(1):85. doi: 10.1186/s12887-018-1026-x . 29475433 PMC5824554

[61] Meesters N, Simons S, Van Rosmalen J, Reiss I, Van Den Anker J, Van Dijk M. Waiting 2 minutes after sucrose administration-unnecessary? Arch Dis Child Fetal Neonatal Ed. 2017;102(2):F167–F9. doi: 10.1136/archdischild-2016-310841 . 28157669 PMC5339570

[62] Bueno M, Yamada J, Harrison D, Khan S, Ohlsson A, Adams-Webber T, et al. A systematic review and meta-analyses of nonsucrose sweet solutions for pain relief in neonates. Pain Res Manag. 2013;18(3):153–61. doi: 10.1155/2013/956549 . 23748256 PMC3673933

[63] Perry M, Tan Z, Chen J, Weidig T, Xu W, Cong XS. Neonatal Pain: Perceptions and Current Practice. Crit Care Nurs Clin North Am. 2018;30(4):549–61. doi: 10.1016/j.cnc.2018.07.013 . 30447813 PMC6570422

