# Supplementary material for: Efficacy and safety of combined oral sucrose and nonnutritive sucking in pain management for infants: A systematic review and meta-analysis
Source: PLoS One. 2022 May 6;17(5):e0268033. doi: 10.1371/journal.pone.0268033 (PMC9075656; doi:10.1371/journal.pone.0268033)
Supplement: S1 Table — (DOCX) [file pone.0268033.s002.docx]

S1 table. Full searching strategy (From Jan 1, 2000 to Mar 31, 2021)

| 50 records were identified via Pubmed (Medline): |
| --- |
| (newborn[MeSH] OR newborn OR premature OR infan* OR neonat*) AND (nonnutritive sucking[Title/Abstract] OR non-nutritive sucking[Title/Abstract] OR NNS[Title/Abstract]) AND (sucrose[Title/Abstract]) AND (pain OR procedural pain OR pain management) AND (randomized controlled trial [pt] OR controlled clinical trial [pt] OR randomized [tiab] OR placebo [tiab] OR clinical trials as topic [mesh: noexp] OR randomly [tiab] OR trial [ti]) NOT (animals [mh] NOT humans [mh]) |
|  |
|  |
|  |
|  |
| 65 Trials identified in Cochrane library |
| newborn OR premature or infan* or neonat* in All Text AND nonnutritive sucking OR non-nutritive sucking OR NNS in Title Abstract Keyword AND sucrose in Title Abstract Keyword AND pain OR procedural pain OR pain management in All Text with Cochrane Library publication, in Trials (Word variations have been searched) |
|  |
|  |
|  |
|  |
| 63 records were identified via OVID (Embase) |
| (newborn or premature or infan* or neonat*).af. and (nonnutritive sucking or non-nutritive sucking or NNS).ab. and sucrose.ab. and (pain or procedural pain or pain management).af. and (randomized controlled trial or controlled clinical trial or randomized or placebo or clinical trials as topic or randomly or trial or clinical trial).af. and (human not animal).af. |
|  |
|  |
|  |
|  |
| 52 records were identified in Web of Science |
| TS=(newborn or premature or infan* or neonat*) and AB=(nonnutritive sucking or non-nutritive sucking or NNS) and AB=sucrose and TS=(pain or procedural pain or pain management) AND TS=(randomized controlled trial or controlled clinical trial or randomized or placebo or clinical trials or randomly or trial or clinical trial)  Indexes=SCI-EXPANDED, SSCI, A&HCI, CPCI-S, CPCI-SSH, BKCI-S, BKCI-SSH, ESCI, CCR-EXPANDED |
|  |
|  |
|  |
|  |
