# Supplementary material for: Efficacy and safety of combined oral sucrose and nonnutritive sucking in pain management for infants: A systematic review and meta-analysis
Source: PLoS One. 2022 May 6;17(5):e0268033. doi: 10.1371/journal.pone.0268033 (PMC9075656; doi:10.1371/journal.pone.0268033)
Supplement: Attachment — Submitted filename: Response to Reviewers.doc [file pone.0268033.s003.doc]

**Response letter**

**Dear Editors and Reviewers,**

**Thank you for your valuable comments. We have revised them one by one according to these comments. We hope that the revised manuscript is now acceptable for publication in your journal.**

**Thank you again for your time and consideration.**

Reviewers' comments:

Reviewer's Responses to Questions

**Comments to the Author**

1. Is the manuscript technically sound, and do the data support the conclusions?

The manuscript must describe a technically sound piece of scientific research with data that supports the conclusions. Experiments must have been conducted rigorously, with appropriate controls, replication, and sample sizes. The conclusions must be drawn appropriately based on the data presented.

Reviewer #1: Yes

Reviewer #2: Partly

2. Has the statistical analysis been performed appropriately and rigorously?

Reviewer #1: Yes

Reviewer #2: No

3. Have the authors made all data underlying the findings in their manuscript fully available?

The [PLOS Data policy](http://www.plosone.org/static/policies.action#sharing) requires authors to make all data underlying the findings described in their manuscript fully available without restriction, with rare exception (please refer to the Data Availability Statement in the manuscript PDF file). The data should be provided as part of the manuscript or its supporting information, or deposited to a public repository. For example, in addition to summary statistics, the data points behind means, medians and variance measures should be available. If there are restrictions on publicly sharing data—e.g. participant privacy or use of data from a third party—those must be specified.

Reviewer #1: Yes

Reviewer #2: Yes

4. Is the manuscript presented in an intelligible fashion and written in standard English?

PLOS ONE does not copyedit accepted manuscripts, so the language in submitted articles must be clear, correct, and unambiguous. Any typographical or grammatical errors should be corrected at revision, so please note any specific errors here.

Reviewer #1: Yes

Reviewer #2: No

5. Review Comments to the Author

Please use the space provided to explain your answers to the questions above. You may also include additional comments for the author, including concerns about dual publication, research ethics, or publication ethics. (Please upload your review as an attachment if it exceeds 20,000 characters)

Reviewer #1: Interesting, well written and well designed review and meta--analysis on efficacy and safety of non-pharmacological interventions on pain relief in infants.

Here my comments:
- introduction:once you introduce the abbreviation e.g OS, I suggest to use it instead of repeating oral sucrose.

*Reply: Thanks. This problem has been updated.*
- methods: please clarify if the research was restricted for english language articles or not; the statements were not clear for this point.

*Reply: “Non-English literature were excluded.” was clarified in the exclusion criteria.(page 5 line 97)*
- discussion: you were discussing about non pharamcological intervention; I think that citing studies about ketorolac was unnecessary.

*Reply: Thanks for your suggestion. The references and related opinions have been deleted.*

Reviewer #2: The authors have made great efforts in doing this systematic review and meta-analysis. There are few concerns :
1. How was the cross over trial managed and which data was included in the meta-analysis is not mentioned. The methods section should clearly indicate how the cross-over designs will be dealt with.

*Reply: The results of cross-over design trials were standardized. The details were added to the method section. (page 7 line 130)*
2. The forests plots show critical levels of heterogeneity despite the subgroup analysis. The manuscript doesn't describe any efforts to explore the reasons for this heterogeneity. It is also worth considering if the meta-analysis with such high level of heterogeneity is required. If yes please add few lines about exploring heterogeneity. It is acceptable to have unexplained heterogeneity but a justification and reasoning with references is required.

*Reply: Possible explanations of high heterogeneity have been added to the limitation part. (page 17 line 342)*
3. Authors have interpreted the funnel plot as showing publication bias. It would be better to add Eggers test and then describe the funnel plots. Only consider funnel plots if number of studies in an analysis are more then 10

*Reply: Thanks for your advice. Egger’s Test has been added. (page 12 line 235)*
4. Authors have mentioned "sensitivity analysis conducted on 2 aspects..." please describe these 2 aspects and also add some details about sensitivity analysis in the method section

*Reply: Two aspects were described(page 10 line 192). Some sentences about sensitivity analysis were added to the last paragraph in method section. (page 7 line 148)*
5. Overall a relook at the grammar and rephrasing of certain sentences is advised.

*Reply: Thank you. The whole manuscript has been updated.*

6. PLOS authors have the option to publish the peer review history of their article ([what does this mean?](https://journals.plos.org/plosone/s/editorial-and-peer-review-process#loc-peer-review-history)). If published, this will include your full peer review and any attached files.


If you choose “no”, your identity will remain anonymous but your review may still be made public.


**Do you want your identity to be public for this peer review?** For information about this choice, including consent withdrawal, please see our [Privacy Policy](https://www.plos.org/privacy-policy).

Reviewer #1: No

Reviewer #2: No

[NOTE: If reviewer comments were submitted as an attachment file, they will be attached to this email and accessible via the submission site. Please log into your account, locate the manuscript record, and check for the action link "View Attachments". If this link does not appear, there are no attachment files.]

While revising your submission, please upload your figure files to the Preflight Analysis and Conversion Engine (PACE) digital diagnostic tool, <https://pacev2.apexcovantage.com/>. PACE helps ensure that figures meet PLOS requirements. To use PACE, you must first register as a user. Registration is free. Then, login and navigate to the UPLOAD tab, where you will find detailed instructions on how to use the tool. If you encounter any issues or have any questions when using PACE, please email PLOS at [figures@plos.org](mailto:figures@plos.org). Please note that Supporting Information files do not need this step.

**Finally, we would like to express my sincere appreciations to you for your hard work, dear editors and reviewers. We look forward to having more opportunities to learn from you in the future!**

**Best regards!**
